# Marine reserves shape seascapes on scales visible from space

**DOI:** 10.1098/rspb.2019.0053

**Published:** 2019-04-24

**Authors:** Elizabeth M. P. Madin, Alastair R. Harborne, Aaron M. T. Harmer, Osmar J. Luiz, Trisha B. Atwood, Brian J. Sullivan, Joshua S. Madin

**Affiliations:** 1 Department of Biological Sciences, Macquarie University, Sydney, New South Wales 2109, Australia; 2 Hawaii Institute of Marine Biology, University of Hawaii, Manoa, HI 96744, USA; 3 Marine Spatial Ecology Laboratory and Australian Research Council Centre of Excellence for Coral Reef Studies, School of Biological Sciences, The University of Queensland, Brisbane, Queensland 4072, Australia; 4 Department of Biological Sciences, Florida International University, 3000 NE 151st Street, North Miami, FL 33181, USA; 5 Institute of Natural and Mathematical Sciences, Massey University, Auckland 0745, New Zealand; 6 Research Institute for the Environment and Livelihoods, Charles Darwin University, Darwin, Northern Territory, Australia; 7 Global Change Institute, University of Queensland, St Lucia, Queensland, Australia; 8 Department of Watershed Sciences and Ecology Center, Utah State University, Logan, UT, USA; 9 Google Earth Outreach, Google, Mountain View, CA 94043, USA

**Keywords:** marine reserves, coral reefs, remote sensing, herbivory, species interactions, Great Barrier Reef

## Abstract

Marine reserves can effectively restore harvested populations, and ‘mega-reserves’ increasingly protect large tracts of ocean. However, no method exists of monitoring ecological responses at this large scale. Herbivory is a key mechanism structuring ecosystems, and this consumer–resource interaction's strength on coral reefs can indicate ecosystem health. We screened 1372, and measured features of 214, reefs throughout Australia's Great Barrier Reef using high-resolution satellite imagery, combined with remote underwater videography and assays on a subset, to quantify the prevalence, size and potential causes of ‘grazing halos’. Halos are known to be seascape-scale footprints of herbivory and other ecological interactions. Here we show that these halo-like footprints are more prevalent in reserves, particularly older ones (approx. 40 years old), resulting in predictable changes to reef habitat at scales visible from space. While the direct mechanisms for this pattern are relatively clear, the indirect mechanisms remain untested. By combining remote sensing and behavioural ecology, our findings demonstrate that reserves can shape large-scale habitat structure by altering herbivores' functional importance, suggesting that reserves may have greater value in restoring ecosystems than previously appreciated. Additionally, our results show that we can now detect macro-patterns in reef species interactions using freely available satellite imagery. Low-cost, ecosystem-level observation tools will be critical as reserves increase in number and scope; further investigation into whether halos may help seems warranted. *Significance statement*: Marine reserves are a widely used tool to mitigate fishing impacts on marine ecosystems. Predicting reserves' large-scale effects on habitat structure and ecosystem functioning is a major challenge, however, because these effects unfold over longer and larger scales than most ecological studies. We use a unique approach merging remote sensing and behavioural ecology to detect ecosystem change within reserves in Australia's vast Great Barrier Reef. We find evidence of changes in reefs' algal habitat structure occurring over large spatial (thousands of kilometres) and temporal (40+ years) scales, demonstrating that reserves can alter herbivory and habitat structure in predictable ways. This approach demonstrates that we can now detect aspects of reefs' ecological responses to protection even in remote and inaccessible reefs globally.

## 1. Introduction

As a globally pervasive conservation and ecosystem-based management tool, no-take marine reserves (hereafter ‘reserves’) are increasing in both number and area, with 19 record-breaking ‘mega-reserves’ (more than 100 000 km^2^) established since 2009 [1]. In these reserves, as well as in smaller and less remote reserves, no method currently exists for remotely observing and monitoring effects that reserves have on species interactions over scales of entire seascapes, despite the need for such methods due to the increasing number and scope or reserves worldwide [2]. It therefore remains largely unknown if and how reserves can predictably alter habitat structure over large scales, nor how to track such changes over increasingly large areas.

**Figure 1.**
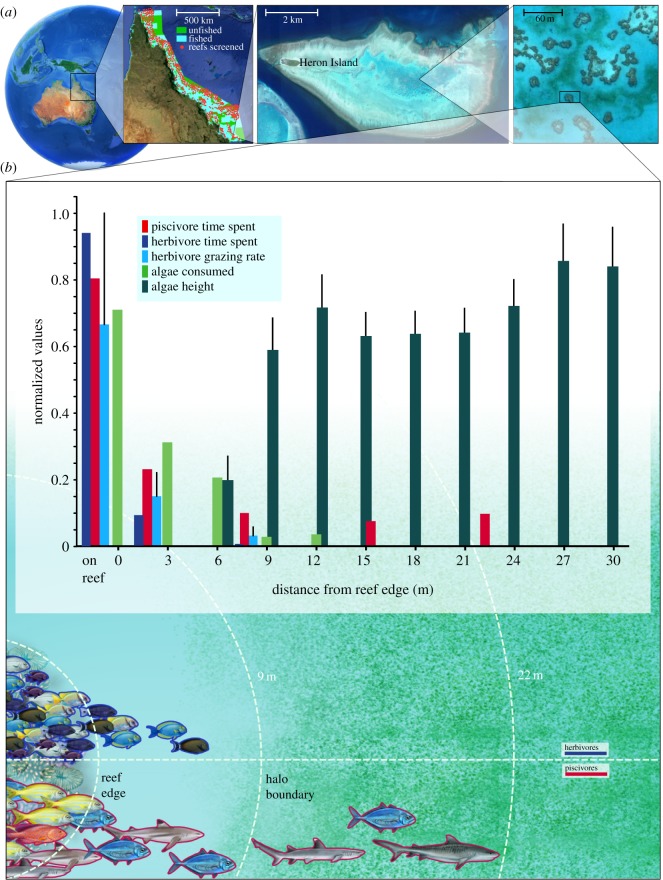
(*a*) Geographical scope and location of study. Along Australia's NE coast, the Great Barrier Reef Marine Park stretches for approximately 2300 km (left square panel) and includes a mosaic of fished areas (blue zones, or ‘General Use Zones’ in which most types of fishing are allowed) and protected, unfished marine reserve areas (green zones, or ‘Marine National Park Zones’ in which fishing is not allowed). Satellite images were used to screen 1372 individual whole reefs (red dots) which span this entire region, including Heron Island (middle panel), where detailed ecological studies of 22 of the lagoon's grazing halos surrounding patch reefs (right panel; dark brown areas are patch reefs, light blue contours around reefs are grazing halos and green zones between halos are algal ‘meadows’) were conducted. Heron Island lagoon is zoned as both no-take (green zone) and limited fishing allowed (yellow zone), but in practice it is essentially unfished (see electronic supplementary material for details). Heron Island lagoon's reserve is considered mature, having been established in 1974 as one of the first two no-take reserves on the Great Barrier Reef. (*b*) Schematic of interaction pathway through which grazing halos are generally believed to occur and evidence from Heron Island lagoon for the role of species interactions in grazing halo formation. Daytime remote video surveys demonstrate that herbivorous fishes spend dramatically more time closer to the shelter and relative safety of the patch reef than in the adjacent sand flat habitat that is devoid of physical structure and thus shelter (dark blue bars). Predators spend more time on and around the reef, but are found throughout the grazing halo and beyond (red bars). Grazing intensity by herbivores is highest close to the reef and drops off precipitously with increasing distance from the reef (light blue bars). By 15 m from the reef, we recorded no grazing by herbivores. Grazing assays conducted over a period of approximately 3 days demonstrate that the per cent of algae consumed by herbivores is functionally absent by 9 m from the reef and beyond (light green bars). The density of algae, measured as canopy height, rises significantly with increasing distance from the reef (dark green bars). All values are normalized for simplicity; *y*-axis maximum values are: 0.42 for herbivore and piscivore time spent (proportion); 0.8 for herbivore grazing rate (bites/min); 100 for algal canopy height (mm) and algae consumed (%). Remote video surveys were conducted in both fished and unfished zones of Heron Island lagoon, however fishing pressure within the lagoon is negligible. Herbivore and piscivore icons are not to scale nor comprehensive, though graphical distributions are qualitatively representative of observed patterns. Herbivore bite rate and time spent data redrawn from Atwood *et al*. [6]; algal canopy height and consumption data are reproduced from Madin *et al.* [7]. Video footage analysis follows protocols outlined in detail in [8].

Many examples of large-scale regular pattern formation in natural ecosystems exist, and the mechanisms behind these patterns are known for some, but relatively few such examples come from marine systems [3]. The well-known phenomenon of ‘grazing halos’ [4,5], which occur on coral reefs worldwide in a variety of primary producer habitats (e.g. benthic macroalgae; seagrass), are landscape-scale vegetation patterns consisting of a halo of heavily grazed, vegetation-free substrate (often ‘sandy white’) surrounding spatially isolated coral patch reefs (figure 1*a*). Grazing halos (hereafter halos) are ecologically significant because they represent a clear, visible indicator of both reefs' ecological functioning (i.e. herbivory and species' behavioural interactions [9]) and habitat structure (vegetation patterns). Furthermore, halos can affect a key ecosystem service: carbon sequestration [6].

The key direct mechanism behind the creation of grazing halos is herbivory. Results from different ocean basins and reef ecosystems have shown that removal of herbivores from a patch reef surrounded by a halo results in the disappearance of the halo [4], that net herbivory declines with increasing distance from reefs [10], and that patterns of herbivory directly match the spatial pattern of algae created in halos across multiple biogeographic regions (figure 1*b*) [7,11,12]. The occurrence of halos therefore demonstrates that reef ecosystems have functional herbivore populations and resultant herbivory, indicating the presence of this critical ecosystem function.

In addition to herbivory, several additional hypotheses have been proposed in the literature or could conceivably account for the direct mechanism(s) behind the formation of halos around patch reefs. These hypotheses include nutrient availability [13], volatile inhibitors of primary producer growth [4–6], bioturbation [13], and physical factors such as sediment particle size [4–7], slope of the benthos [4,5], wave energy [4,7] and shading [7]. These mechanisms are not mutually exclusive with one another, nor with herbivory. For example, in our *in situ* study system of Heron Island lagoon, there is some evidence that bioturbation may contributing to halo formation along with the recognized key direct mechanism of herbivory [8]. Similarly, surface sediment particle size does vary significantly with distance from patch reefs [14] within Heron Island lagoon but was not found to have an effect on algal canopy height over the halo gradient [6]. Each of the hypotheses above been considered in previous studies in this or other study systems and deemed unable, through testing or logic, to account in isolation for the halo patterns observed [4–7,14,15].

The indirect mechanisms governing the spatial patterns of herbivory behind halos are less well understood. Both competition for food resources among herbivores and predation risk imposed on herbivores by their predators could lead to the spatial patterns of grazing that create halos, and these mechanisms are not mutually exclusive. In the first case, herbivore density on isolated patch reefs, and thus competition by both conspecifics and heterospecifics for food resources beyond the reef's edge, should affect the distance that animals need to travel from the reef to obtain sufficient nutrition. Indeed, evidence that herbivore density affects halo width in another system exists [12], though in this case patch reef size covaried with herbivore density, rendering it difficult to disentangle the effects of herbivore density and habitat area on halo width. In the latter case of predation risk shaping herbivory, grazing halos have long been assumed to be the collective result of many small herbivores' (e.g. fishes' and urchins') reluctance to travel far from coral reefs' shelter for fear of consumption by predators in the riskier, unsheltered areas beyond [4,5]—even though these pioneering studies occurred in systems known to have previously experienced significant fishing pressure, predator loss, and presumably reduced predation risk. Foraging theory likewise predicts that areas with greater predation risk, such as those farther from shelter, should experience less resource harvest by mobile animals than areas less exposed to predators [16]. Atwood *et al*. [6] recently confirmed the occurrence of this mechanism in their Heron Island lagoon study location by showing that that predators consistently suppressed foraging by herbivores within halos via risk effects. These effects were manifest specifically through herbivores' grazing rates and time budgets.

We thus hypothesized that, through spatially constrained herbivory due to one or both of these indirect mechanisms, marine reserves could affect halo prevalence and/or size by altering herbivore and/or predator populations. Specifically, if reserves result in greater herbivore density relative to fished reefs, halos would be expected to be more prevalent (if fished areas experienced functional loss of herbivores) and/or larger in reserves. On herbivore-poor reefs in which the complete functional loss of herbivores has occurred, herbivores would not be expected to exert sufficient grazing pressure to create halos [4]. Conversely, on predator-rich reefs, as expected within reserves, herbivorous prey should face substantial predation risk and preferably forage near shelter, with their collective grazing patterns leading to more intense grazing around reefs. In this sense, reserves should lead to more prevalent and/or smaller grazing halos than in areas with fewer predators. On predator-poor reefs, roving herbivores without such constraints might range more widely [17], dispersing their grazing effort more evenly over the seascape [18] and resulting in a widening or loss (the latter due to merging or lack of creation) of halos.

To test these predictions, we combined high-resolution satellite imagery surveys, remote underwater videography, and *in situ* manipulations to assess the spatial extent and potential causes of these large-scale vegetation patterns surrounding coral patch reefs (i.e. spatially isolated, metres-scale reefs). Our objective was to determine if and how marine reserves affect halo prevalence and size in order to further our understanding of the potential for halos to be used as an indicator of aspects of coral reef ‘health’ and ecological functioning.

## 2. Methods

### (a) *In situ* surveys

To confirm the role of the direct mechanism of herbivory and explore the hypothesized indirect mechanism of predation risk in forming halos within the Great Barrier Reef (GBR), we used remote underwater video (camera trap) surveys conducted at a suite of 22 individual patch reefs within the predator-rich marine reserve and adjacent fished zone within Heron Island lagoon encompassing a mature reserve in the southern GBR (see electronic supplementary material for details). Although part of Heron Island lagoon can legally be fished, the geomorphology of the lagoon means that its access points are difficult to navigate and are strongly tidally dependent. As a result, the lagoon experiences negligible fishing pressure, which is primarily on predators, and both fished and unfished parts of the lagoon show similar patterns in predator and herbivore abundance and behaviour relative to patch reefs (electronic supplementary material, figure S1).

Camera trap surveys (via continuous video) were conducted at 22 patch reefs/halos during daytime hours over three weeks in May 2013 and consisted of placing arrays of GoPro high-resolution video cameras on patch reefs and within the grazing halos. At each reef, one camera was placed atop the reef and four cameras were placed along a linear transect radiating outwards (into and beyond the halo) at distances of 2.5, 7.5, 15 and 22 m from the reef's edge. Post-processing of video data involved classifying all individual non-cryptic fish and invertebrate species recorded to species level (which was possible in most cases) or, where not possible, to genus or family level. The duration of time the organism was within the observation area was also recorded. Plant-eating fishes were classified as ‘herbivores’ and fish-eating predatory fishes were classified as ‘piscivores’. All species known to fall under these categories were included in analyses unless otherwise noted. Further details can be found in the electronic supplementary material.

### (b) Remote sensing surveys

To test our predictions regarding halo presence and size, we used high-resolution satellite imagery available in the Google Earth Pro platform (see electronic supplementary material for details) to screen for halos surrounding patch reefs (i.e. small, spatially-separated reefs with diameters of metres to tens of metres) located within 1372 whole reefs. Whole reefs are kilometre-scale reef ecosystems that are separated from one another by deeper water, within which tens to thousands of individual coral patch reefs can be found. We systematically screened the approximately 2300 km latitudinal gradient of Australia's Great Barrier Reef Marine Park (figure 1*a*) containing thousands of whole reefs, of which approximately 33% are reserves and approximately 67% are fished. To collect these data, cross-continental shelf transects from the Australian coastline to the seaward extent of the GBR were scanned at every 0.05° of latitude from −10.7° to −24.5°. When a whole reef was encountered along a transect, its name, latitude, longitude, areas, fishing status, image quality and image date were recorded. If an individual whole reef was encountered on multiple transects (e.g. as was the case with many north–south oriented ribbon reefs) it was only surveyed once. Only imagery that was of less than or equal to 3 m spatial resolution was used in this study because the spatial scale of halos dictates that only at this resolution can they be reliably identified and measured. Our screening resulted in 214 focal whole reefs with both suitable habitat for halos and adequate image quality with which to visually detect them (72 in reserves and 142 in fished areas; figure 1*a*; see electronic supplementary material).

Each of our 214 focal whole reefs contains hundreds to thousands of individual patch reefs (separated from one another by expanses of shallow, flat habitat) around which halos can form. Grazing halo presence at the whole reef level was scored as ‘halos present’ if any patch reefs within a whole reef were surrounded by halos. Grazing halo size was measured as width, via five replicate linear, equidistant, spoke-like measurements from the outer edge of the patch reef to the edge of the halo, which were then averaged to generate a halo width value for each individual grazing halo. At each whole reef where halos were visible, ten replicate patch reefs/halos were measured (except in cases where halos were visible but fewer than ten could be measured, in which case all halos were measured). Ten was chosen as our number of replicates because (i) this sample size balanced the trade-off between time constraints and sample size, and (ii) variation in halo width appeared visually to be minimal within locations, suggesting to us that more than 10 replicates would not appreciably decrease within-location variability. All halos were measured manually by a single observer (AH). Of the 214 focal reefs, 68 contained halos, and the remaining reefs were scored as halos being absent.

GBR spatial data layers (i.e. zoning, whole reef names/numbers, and other metadata; available from the Great Barrier Reef Marine Park Authority upon request) were imported into GEP. These layers were used to identify whole reef area and protection status (i.e. fished versus protected). Reefs were classified as ‘no-take reserves’ (hereafter reserves) if they were in any of the Great Barrier Reef Marine Park zones in which no commercial or recreational fishing is allowed. These zones are Marine National Park Zone (green zone), Preservation Zone (pink zone) and Scientific Research Zone (orange zone). All other zones (Buffer Zone; Conservation Park Zone; Habitat Protection Zone; General Use Zone) allow some degree of commercial and/or recreational fishing and were therefore classified as ‘fished’. For the reserve age analysis, fished reefs were assigned a reserve age of 0, thereby equating them with a newly-established reserve that had received no prior protection from fishing. Reserve ages did not systematically vary with latitude because reserve designations initially occurred sequentially by region (between 1979 and 1987), but the order of regions designated with reserves did not follow a geographical gradient. Subsequent re-zonation of reserves (in 2004) occurred simultaneously over the entire GBR.

### (c) Variable selection and statistical analyses

We gathered GBR-wide data from existing datasets for all hypothesized factors expected to influence grazing halo occurrence and/or width. We then examined pair-wise interactions between all variables that could plausibly covary. In the absence of available *in situ* herbivore or piscivore density data for any substantial portion of the 214 whole reefs from our satellite imagery dataset that were used in analyses, we included the best-available potential proxies over this large spatial scale (i.e. marine reserve age and distance from mainland, a proxy for human visitation and thus presumed fishing pressure). Other variables included in or considered for the initial model were those hypothesized to affect grazing halo presence and/or width in another way and included: sea surface temperature (SST; a proxy for metabolic rates of herbivores); latitude (both a presumed proxy for metabolic rates of herbivores (via temperature) and a proxy for the biogeographic gradient over which the GBR occurs); chlorophyll a concentration (Chl *a*; a proxy for primary productivity); image season (i.e. the time of year in which the satellite image was captured, to account for seasonal differences in algal standing stock and thus halo visibility); and patch reef area (hypothesized for either geometric and/or biological reasons to potentially influence grazing halo size).

A generalized linear model (function *glm* from R package *stats*) was used to quantify relationships between grazing halo presence and the suite of explanatory variables remaining after the above filtering for suitability (see electronic supplementary material) was completed. As described above, only whole reefs that contained suitable habitat and for which clear satellite imagery was available were included in presence/absence analyses. This ensured that only whole reefs that had the potential to have grazing halos, but did not, were compared with whole reefs that did exhibit halos. Based on the procedures for model selection criteria and identifying interactions among explanatory variables described in Zuur *et al*. [19], the variables that remained in the final model were: marine reserve age, temperature and Chl *a* concentration (electronic supplementary material, table S1). A linear mixed-effects model fit by maximum likelihood (function *lme* from R package *nlme*) was used to quantify relationships between grazing halo width and the same suite of variables above plus grazing halos' interior patch reef area. Because grazing halo and patch reef size data are at the within-whole-reef spatial scale, patch reef area and whole reef ID were included as nested random factors within the model. All other factors, as well as patch reef area, were included as fixed factors. Based on model selection criteria and pair-wise variable interactions [19], the variables that remained in the final model were: marine reserve age, SST, Chl *a* concentration and patch reef area (electronic supplementary material, table S2). All analyses were conducted using the statistical programming package R (R Development Core Team, 2015).

## 3. Results

Our observations within the Heron Island reef system revealed that, as expected based on central place foraging [20] theory, herbivorous fishes throughout the lagoon concentrated their activity within and immediately around reefs, with no herbivores observed beyond 7.5 m from the reef and algal canopy height increasing concomitantly (figure 1*b*, dark blue and green bars, respectively). Specifically, the proportion of time spent by herbivores was concentrated most heavily on the reef itself, where feeding opportunities for mobile browsers (e.g. surgeonfishes; rabbitfishes; some parrotfishes) are limited but spatial refuges from predators exist. Beyond the reef edge, these fishes reduced the amount of time they spent as they moved farther from the reef (figure 1*b*, dark blue bars). This pattern, which is counter to herbivores' expected distribution based on food availability, indicates the existence of a behavioural constraint that spatially restricts their foraging patterns. The maximum distance that by halo-feeding herbivores ventured from the reef was unrelated to their relative abundance (*z* = 1.66, *p* = 0.096; fig. 2A in Madin *et al.* [8]). We found that piscivores likewise concentrated their time on and immediately around the reef, but, unlike herbivores, were also found throughout the halo zone and beyond its boundary into the algal meadows (figure 1*b*; red bars). A list of all fishes and macroinvertebrates observed in our video surveys, and delineation of where species occurred (i.e. on reefs and/or within the halo/meadow zones), is given in electronic supplementary material, table S3.

**Figure 2.**
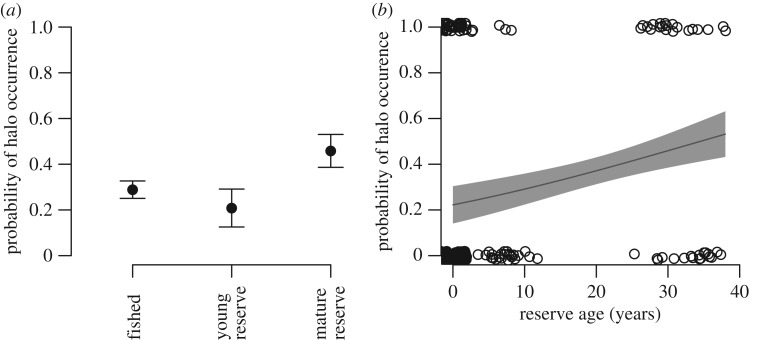
Probability of grazing halo occurrence as a function of (*a*) reserve status and (*b*) no-take marine reserve age. In (*a*), points represent means (±s.e.) of the probability of halo occurrence within whole reefs across the three reserve status categories. ‘Mature’ reserves are those that had passed the threshold at which fisheries-targeted species recovery generally occurs, as shown by Babcock *et al*.'s [21] cross-ecosystem study of marine reserves which found that the upper limit of time to initial detection of direct effects on targeted species was 7.12 (mean 5.13 ± 1.9) years. ‘Young’ reserves are 7 or fewer years old. In (*b*), solid line is model fit; shaded area is standard error. Open points represent whole reefs where grazing halos were observed (value = 1) or not observed (value = 0) as a function of reserve age. Points are jittered to improve visibility. Each whole reef contains hundreds to thousands of individual patch reefs around which grazing halos can potentially form.

Our satellite imagery survey demonstrated that grazing halos are significantly more common within mature (≥8 years old) reserves than on fished reefs (*χ*^2^(1, 190) = 3.92, *p* = 0.048; figure 2*a*). Reserves were deemed ‘mature’ when they had passed the threshold at which fisheries-targeted species recovery generally occurs, as shown by Babcock *et al*.'s [21] cross-ecosystem study of marine reserves which found that the upper limit of time to initial detection of direct effects on harvested species was 7.12 (mean 5.13 ± 1.9) years. From the ten predictor variables initially hypothesized to influence grazing halo prevalence, we used model selection techniques to isolate the most important variables, which were subsequently included in analyses. These variables were reserve age, sea surface temperature (SST; a proxy for metabolic rates), and chlorophyll *a* concentration (Chl *a*; a proxy for primary productivity; see electronic supplementary material).

A generalized linear model (GLM) showed that reserve age was a significant predictor of halo occurrence across the GBR (table 1; electronic supplementary material, table S1; *R*^2^ = 0.072). Specifically, as reserves age, halo occurrence probability increases significantly, with the likelihood of grazing halo occurrence increasing by approximately 250% from a newly established reserve to a 40-year-old reserve (figure 2*b*). The remaining two variables, SST and Chl *a*, were not significant predictors of grazing halo occurrence. We tested for significant pairwise and three-way interactions among the predictor variables and found none.

**Table 1.** AIC values used in model selection process. Best-fit model was selected by using the *drop1* function with chi-square test in R; analogous results were obtained by using a stepwise model selection procedure with R function *step*. For grazing halo presence analyses, a generalized linear model was used (R function *glm*); for grazing halo size model, a linear mixed effects model was used (R function *lme*). Patch reef area could not be included in the ‘Halo presence’ model because halo presence was measured at the whole-reef scale, whereas patch reef area was measured at the within-whole-reef scale. Italics indicate significance at *p* < 0.05.

	halo presence		halo size	
model/variable	model AIC when dropped	*p*-value	model AIC when dropped	*p*-value
null	95.510	n.a.	−149.46	n.a.
reserve age	97.919	*0.036*	−150.31	0.285
sea surface temperature	94.450	0.332	−146.27	*0.023*
chlorophyll *a* concentration	96.856	0.067	−151.45	0.988
patch reef area	n.a.	n.a.	−134.88	*4.66 × 10^−5^*

**Figure 3.**
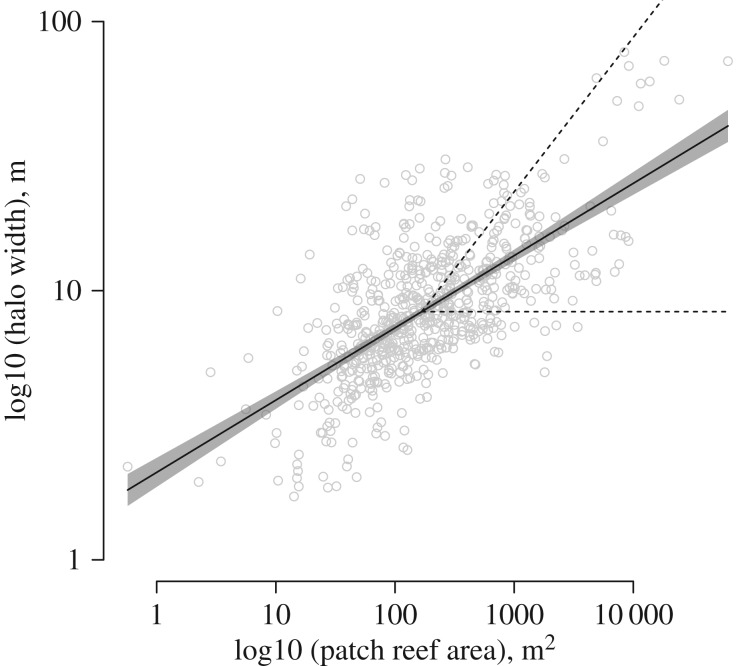
Halo width as a function of patch reef area. Points are individual patch reefs/halos from across the GBR; solid line is linear model fit; shaded area is 95% confidence interval. Dashed lines are null model slopes for expected relationships arising from a perimeter-based (lower line of slope = 0) or area-based (upper line of slope = 0.5) effect of patch reef area on halo width.

Contrary to our initial hypothesis, halo size (average width) across the GBR was not significantly related to marine reserve status (fished versus reserve) nor reserve age, but rather was best explained by two environmental factors: patch reef area (i.e. area of reef around which halos form; figure 3) and sea surface temperature (SST). By far the greatest predictor of these two significant predictors of halo width was patch reef area (tables 1 and 2; electronic supplementary material, table S2; marginal *R*^2^ = 0.482; conditional *R*^2^ = 0.617), which explained approximately 71% of the total predictor variation.

**Table 2.** Hierarchical partitioning of variance explained by final model parameters for response variable halo size. Values obtained from R function *hier.part*.

variable	% of total variance explained
patch reef area	71.09
sea surface temperature	20.22

To test whether the slope of the halo width–patch reef area relationship deviated from what would be expected based purely on geometrically based processes, we considered two null models (figure 3, dashed lines) based on the simplifying assumptions that per capita herbivore density and energetic needs are constant, and that reefs are a given and simple shape (circles). An example of a perimeter-based process is herbivory that occurs only by herbivores living at the perimeter of the patch reef, whereas an example of an area-based process is herbivory by herbivores living throughout the full area of the patch reef. The lower dashed line in figure 3 represents the expected relationship between halo width and patch reef area based solely on increased reef perimeter size, such that as reef area increases, halo width remains constant as a function of perimeter (slope = 0). The upper dashed line in figure 3 represents the expected relationship between halo width and patch reef area based solely on increased reef area, such that as reef area increases, halo width increases as a function of that area (slope = 0.5).

## 4. Discussion

Our findings collectively demonstrate that reserves have the capacity to predictably alter the functional importance of herbivores [9], shaping algal habitat structure over large scales as a result. Specifically, in agreement with previous theoretical work [18] and empirical studies from the Caribbean Sea [4,5,22] and Indian Ocean [11], our *in situ* studies show that herbivorous fishes graze benthic macroalgae in a spatially constrained manner (figure 1*b*, blue bars) that forms grazing halos (figure 1*b*, green bars). In addition, our data show that this constrained foraging pattern occurs within a landscape at Heron Island lagoon in which risk of predation increases with distance from reef refugia, given piscivores' presence throughout the reef, halo and algal meadow zones (figure 1*b*, red bars). It has been shown at the same study sites that herbivores' spatial foraging pattern is not likely due to within-guild competition (fig. 2A in Madin *et al.* [8]). Our satellite image surveys provide large spatial and temporal scale evidence that as reserves age, reef ecosystems are increasingly likely to exhibit halo-like patterns (figure 2). Furthermore, individual grazing halos, of which we estimate there to be millions within the GBR alone, occur over a spatial scale large enough (i.e. hundreds to thousands of m^2^ per halo) to be easily viewed from space by anyone via Google Earth's freely available satellite imagery. Halos are also known to affect carbon sequestration because sedimentary carbon accumulation scales with long-term surface macroalgal biomass [6], highlighting halos' potential importance in regulating ecosystem-scale processes. These seascape-scale patterns are repeated throughout the approximately 2300 km of the GBR, and indeed the world (E. Madin 2018, unpublished data; C. Roelfsema 2018, personal communication), demonstrating the geographically widespread extent of a key ecological function (i.e. herbivory) that reserves encompass—as well as its resulting footprint on reef ecosystems.

Our unexpected finding that halo width across the GBR was driven primarily by patch reef area (figure 3) and was not significantly related to marine reserve status (fished versus reserve) nor reserve age can be explained by two possible factors. These explanations differ based on the limiting factors of herbivore population size. First, if space (e.g. for shelter or territories) is the limiting factor for herbivores, larger patch reefs may support higher herbivore densities by virtue of being more structurally complex with greater inhabitable volume, and thus supporting higher densities of herbivores per unit horizontal area, than smaller-area reefs. Conversely, if food is the limiting factor for herbivores, smaller patch reefs should have narrower halos by virtue of geometry. As patch reefs increase in size, their area:perimeter ratio increases. More interior area dictates that more fishes must forage in functionally less perimeter space as patch reef size increases, resulting in wider halos due to the increased impact of more fishes on a smaller perimeter space. These two explanations are not mutually exclusive; both may be at play.

Regardless of the limiting factor (space or food) of herbivore population size, figure 3 offers insight into the type of process (i.e. area- or perimeter-based) that governs the observed relationship between patch reef area and halo width. The slope of this relationship (slope = 0.268) lies between the slopes expected for these two types of processes, specifically that that as reef area increases, halo width remains constant as a function of perimeter (slope = 0) and that as reef area increases, halo width increases as a function of that area (slope = 0.5). Figure 3 therefore demonstrates that halo width is likely to be dictated by a mixture of both perimeter-based and area-based processes.

The other significant predictor variable of grazing halo width was SST. SST had a positive effect on halo width (i.e. areas of higher SST have larger halos). Our *post hoc* interpretation of the SST–halo width relationship is that it is largely a function of the variation in patch reef size over latitude, which is positively correlated with temperature. Specifically, larger patch reefs are on average found in the northern GBR (i.e. at lower latitudes), where temperatures are higher than the more southerly GBR reefs. Thus, larger halos are found in areas with higher temperatures. We tested the relationship between patch reef size and SST *a priori* during the model selection process to determine if the relationship was strong enough such that both variables should not be included in the model, but it was not (Pearson's product-moment coefficient *r* = 0.380, d.f. = 673, *p*-value < 2.2 × 10^−16^). It is also plausible that the increased metabolic rates, and thus energy intake requirements, expected of herbivores under higher temperatures could lead to higher net grazing rates, and thus halo widths, as temperature increases, though a recent meta-analysis of global herbivore impacts on resources casts doubt on this explanation [23]. However, we have not ruled out other alternative explanations for the SST–halo width relationship, and these explanations remain speculative. Of the remaining non-significant predictors, reserve age and Chl *a* both explained less than 5% of the variation in the final model. However, the effect of reserve age on halo width was in the direction predicted by our original hypotheses (i.e. that whole reefs within older reserves should have smaller halos). Whatever effect that reserve age has on halo width was overwhelmed by the large portion of variation explained by the environmental factors in the model. Collectively, our results suggest that halo size results in large part from the interaction between herbivore abundance and behaviour and habitat size and configuration (i.e. patch reef area and shape). Collectively, these results (figures 1 and 3; table 2) demonstrate that although grazing halo occurrence is driven by spatially constrained herbivory, halo size is driven in part by other factors that include reef ecosystems' environmental characteristics.

We did not attempt to distinguish between the relative importance of the indirect (secondary) mechanisms governing herbivory over the scale of the GBR because the data required to do so at this very large scale do not exist. Rather, we focus here on the role of marine reserves in shaping patterns of vegetation through herbivory over large spatial scales. Nonetheless, our *in situ* data from Heron Island lagoon, combined with previous and emerging results from other studies, may offer some insight from individual locations into the likelihood of these mechanisms' roles in generating the GBR-wide patterns we describe.

Specifically, these data collectively allow exploration of the two potentially viable indirect mechanisms (i.e. herbivore competition and predation risk) behind the spatially constrained patterns in herbivory observed within Heron Island lagoon. Herbivore density data from the same suite of reefs (fig. 2A in Madin *et al.* [8]) demonstrates that within-guild density had no clear effect on the maximum distance that herbivorous fishes ventured beyond the patch reefs. It is impractical to remove predators to isolate the role of risk at the relevant ecological scale to determine if predation risk could alternatively impose the observed spatial constraint on herbivory. Instead, our risk titrations (figure 1*b*, light green bars; reproduced from Madin *et al.* [7]) instead allow us to gauge fishes' perception of where the risk of predation outweighs the energetic reward of the food patch over the spatial gradient of the halo [24]. Our results show that as distance from reef shelter and thus predation risk due to large, mobile reef predators increase (figure 1*b*, red bars), net grazing rate declines (figure 1*b*, light green bars). This phenomenon is well documented; large sand gaps between reefs, such as those in our study system, are known to impede fish movements [25] and hence preclude grazing between reefs. In agreement with Atwood *et al*. [6], these results collectively suggest that, at least within Heron Island lagoon, predation risk is a more parsimonious explanation for the observed patterns of herbivory than is herbivore competition.

Whether these patterns extend to other reefs within the wider GBR region and can explain the disparity in halo occurrence in fished versus mature reserve areas (figure 2) is unclear. One way to explore the likely role(s) of the two plausible indirect mechanisms (i.e. herbivore competition and predation risk) in generating the GBR-wide patterns we describe (figure 2) is by considering whether herbivores and/or piscivores are likely to differ in abundance between GBR management zones. Mobile herbivores have been occasionally seen to be greater in biomass or density within individual no-take reserves in the GBR [26,27], though only one of these studies found this to be the case across more than one herbivore species [27], and this was the case in only one of the two regions studied. More often, herbivores have been observed to be less abundant or not significantly different in GBR no-take reserves relative to fished reefs [28,29]. These studies demonstrate that while consistent differences in mobile herbivore assemblages are not seen within or among studies of GBR reserve effects, one cannot eliminate the possibility that individual GBR reserves may harbour higher densities of mobile herbivores than adjacent fished areas. It is also important to recognize that herbivores are not generally targeted by fishers in the GBR [29,30] as they are in many other locations around the world. In terms of piscivores, reserve age and shark density are positively correlated across the approximately 2300 km length of the GBR [31]. Similarly, other predatory fishes (both primary and secondary fisheries target species) have been shown to consistently increase in biomass or density in GBR reserves relative to fished areas [28–30,32]. In another coral reef system, a combination of theoretical and empirical work undertaken throughout a gradient of piscivore density spanning hundreds of kilometres revealed that predation risk was the key mechanism leading to the same pattern of spatially constrained foraging by coral reef fishes that form halos [17,18].

In summary, our local-scale empirical results from Heron Island lagoon, in conjunction with past studies of halos [4,5,7,10,11,25,33,34], past studies of GBR reserve predator and herbivore abundances [28–32], and the fact that herbivores are not heavily targeted by fisheries in the GBR [29,30], suggest that predation risk is a more likely explanation than herbivore competition as the dominant indirect mechanism behind the pattern of halo prevalence across GBR reserves that we describe. Therefore, while it is possible that herbivore density plays a role in governing the size of halos in the GBR as it does elsewhere [12], herbivore density is a less likely explanation of the GBR-wide patterns of marine reserve halo occurrence we describe. Given the global prevalence of halos on reefs, further studies aimed at disentangling the role(s) of predation risk and herbivore competition in indirectly driving halo formation will help elucidate how pervasive these mechanisms are in natural ocean ecosystems. Understanding the extent to which these mechanisms may structure reef ecosystems over large spatial and temporal scales is important, particularly given that fishing patterns—and therefore the indirect mechanisms behind halo formation—may well differ among locations.

Remote observation of grazing halos may allow development of a conservation and ecosystem-based management tool, since halos provide information about if and how reserves are affecting aspects of the functional importance of herbivores, possibly predators, and at least one aspect of reef habitat structure (i.e. vegetation coverage). Furthermore, in fished areas, remotely observing grazing halo presence may indicate if fishing is altering reef ecosystems, for example where harvest intensity changes over time or space. Importantly, grazing halos have been described from a wide range of biogeographical regions [4,5,11,13,22,35–38], demonstrating that this type of analysis may be informative for marine reserve managers in many other locations globally. This method has many benefits: it is low-cost, requires minimal technical expertise/training, covers large areas and allows observers to look back in time where historical imagery exists. Despite its clear benefits, our model of halo occurrence has important limitations. Importantly, although reserve age and halo presence are significantly correlated, the model's low *R*^2^ value (0.072) means that its predictions must be interpreted with caution. For example, our data (figure 2*b*) show that in many marine reserves, including mature reserves, halos may not be apparent. Conversely, many fished reefs may have halos. For this reason, halo presence cannot be considered a litmus test of marine reserve effectiveness, nor of fisheries-induced loss of ecosystem function. Further research into the full suite of mechanisms driving halo formation, and any geographical differences in these mechanisms, will probably increase halos' utility for ecosystem monitoring. Additionally, monitoring coral reefs remotely is not a replacement for traditional *in situ*, diver-based ecological surveys. The two methods must be used in concert, with remote observation providing a broad-brush picture of ecosystem habitat structure (and aspects of ecosystem function), and *in situ* monitoring used to ground-truth and calibrate remote assessments. Importantly, *in situ* scientific surveys are required to understand the ecological details of reef ecosystems, from which we can then make informed interpretations of data gathered from remote surveys. Our work shows that the technology for remotely observing reefs already exists. The main limitation in applying this method universally is the lack of globally comprehensive, high-frequency, very high-resolution satellite imagery, for example to determine the temporal scale over which halos appear/disappear and/or change in size. This imagery could potentially be obtained for approximately US$1 million [39] (see electronic supplementary material), a cost that should fall rapidly as nano-satellites become more abundant and their imagery more accessible [40]. For example, near-daily imagery of much of the globe now exists at approximately 3 m spatial resolution. Though this resolution is too coarse for quantifying changes in halo size, it does allow for detection of halo presence and will probably improve in the near future [41]. To quantify effectiveness of the world's marine reserves, we must look beyond current methods to more scalable, cost-effective approaches [2]—of which satellite-based observation of the world's reefs may be one.

## Supplementary Material

Supplementary information

## Data accessibility

All data and code used in this study are available for download at 10.5281/zenodo.2602077.

## References

[1] McCauley DJ, Woods P, Sullivan B, Bergman B, Jablonicky C, Roan A, Hirshfi M, Boerder K, Worm B 2016 Ending hide and seek at sea. Science 351, 1148–1150. (10.1126/science.aad5686) 26965610

[2] McCauley DJ, Morgan L, Possingham H, White L, Darling E, Jones PJS 2014 Mega-parks. Nature 515, 28–31. (10.1038/515028a) 25373660

[3] Rietkerk M, van de Koppel J. 2008 Regular pattern formation in real ecosystems. Trends Ecol. Evol. 23, 169–175. (10.1016/j.tree.2007.10.013) 18255188

[4] Ogden J, Brown R, Salesky N 1973 Grazing by the echinoid *Diadema antillarum* Philippi: formation of halos around West Indian patch reefs. Science 182, 715–717. (10.1126/science.182.4113.715) 17817963

[5] Randall JE 1965 Grazing effect on sea grasses by herbivorous reef fishes in the West Indies. Ecology 46, 255–260. (10.2307/1936328)

[6] Atwood TB, Madin EMP, Harborne AR, Hammill E, Luiz OJ, Ollivier Q, Roelfsema C, Macreadie PI, Lovelock CE 2018 Predators shape sedimentary carbon storage in a coral reef ecosystem. Front. Ecol. Evol. 6, 110 (10.3389/fevo.2018.00110)

[7] Madin EMP, Madin JS, Booth DJ 2011 Landscape of fear visible from space. Sci. Rep. 1, 14 (10.1038/srep00014) 22355533 PMC3216502

[8] Madin EMP, Precoda K, Harborne AR, Atwood TB, Roelfsema C, Luiz OJ In press Multi-trophic species interactions shape seascape-scale coral reef vegetation patterns. Front. Ecol. Evol.

[9] Srivastava DS, Vellend M 2005 Biodiversity-ecosystem function research: is it relevant to conservation? Annu. Rev. Ecol. Evol. Syst. 36, 267–294. (10.1146/annurev.ecolsys.36.102003.152636)

[10] Gil MA, Zill J, Ponciano JM 2017 Context-dependent landscape of fear: algal density elicits risky herbivory in a coral reef. Ecology 98, 534–544. (10.1002/ecy.1668) 27870010

[11] Downie RA, Babcock RC, Thomson DP, Vanderklift MA 2013 Density of herbivorous fish and intensity of herbivory are influenced by proximity to coral reefs. Mar. Ecol. Prog. Ser. 482, 217–225. (10.3354/meps10250)

[12] DiFiore BP, Queenborough SA, Madin EMP, Paul VJ, Decker MB, Stier AC In review Predation risk and landscape features drive variation in grazing patterns visible from space. Mar. Ecol. Prog. Ser.

[13] Alevizon W 2002 Enhanced seagrass growth and fish aggregations around Bahamian patch reefs: the case for a functional connection. Bull. Mar. Sci. 70, 957–966.

[14] Ollivier QR, Atwood TB, Booth DJ, Hinchliffe C, Madin EMP, Harborne AR, Lovelock CE, Macreadie PI, Hammill E 2018 Benthic meiofaunal community response to the cascading effects of herbivory within an algal halo system of the Great Barrier Reef. PLoS ONE 13, e0193932 (10.1371/journal.pone.0193932) 29513746 PMC5841801

[15] Bartholomew B 1970 Bare zone between California shrub and grassland communities: the role of animals. Science 170, 1210–1212. (10.1126/science.170.3963.1210) 17744053

[16] Brown JS 1999 Vigilance, patch use and habitat selection: foraging under predation risk. Evol. Ecol. Res. 1, 49–71.

[17] Madin EMP, Gaines SD, Warner RR 2010 Field evidence for pervasive indirect effects of fishing on prey foraging behavior. Ecology 91, 3563–3571. (10.1890/09-2174.1) 21302828

[18] Madin EMP, Gaines SD, Madin JS, Warner RR 2010 Fishing indirectly structures macroalgal assemblages by altering herbivore behavior. Am. Nat. 176, 785–801. (10.1086/657039) 20961223

[19] Zuur AF, Ieno EN, Walker NJ, Saveliev AA, Smith GM 2009 Mixed effects models and extensions in ecology with R. New York, NY: Springer.

[20] Orians GH, Pearson NE 1979 On the theory of central place foraging. In Analysis of ecological systems (eds Horn DJ, Stairs GR, Mitchell RD), pp. 155–177. Columbus, OH: Ohio State University Press.

[21] Babcock RC, Shears NT, Alcala AC, Barrett NS, Edgar GJ, Lafferty KD, McClanahan TR, Russ GR 2010 Decadal trends in marine reserves reveal differential rates of change in direct and indirect effects. Proc. Natl Acad. Sci. USA 107, 18 256–18 261. (10.1073/pnas.0908012107) PMC2972978 20176941

[22] Armitage AR, Fourqurean JW 2006 The short-term influence of herbivory near patch reefs varies between seagrass species. J. Exp. Mar. Bio. Ecol. 339, 65–74. (10.1016/j.jembe.2006.07.013)

[23] Poore AGB et al. 2012 Global patterns in the impact of marine herbivores on benthic primary producers. Ecol. Lett. 15, 912–922. (10.1111/j.1461-0248.2012.01804.x) 22639820

[24] Brown JS, Kotler BP 2004 Hazardous duty pay and the foraging cost of predation. Ecol. Lett. 7, 999–1014. (10.1111/j.1461-0248.2004.00661.x)

[25] Turgeon K, Robillard A, Grégoire J, Duclos V, Kramer DL 2014 Functional behavioral a reef fish connectivity from perspective: behavioral tactics for moving fragmented landscape. Ecology 91, 3332–3342. (10.1890/09-2015.1) 21141194

[26] Graham NAJ, Evans RD, Russ GR 2003 The effects of marine reserve protection on the trophic relationships of reef fishes on the Great Barrier Reef. Environ. Conserv. 30, 200–208. (10.1017/S0376892903000195)

[27] Casey JM, Baird AH, Brandl SJ, Hoogenboom MO, Rizzari JR, Frisch AJ, Mirbach CE, Connolly SR 2017 A test of trophic cascade theory: fish and benthic assemblages across a predator density gradient on coral reefs. Oecologia 183, 161–175. (10.1007/s00442-016-3753-8) 27744581

[28] Williamson DH, Russ GR, Ayling AM 2004 No-take marine reserves increase abundance and biomass of reef fish on inshore fringing reefs of the Great Barrier Reef. Environ. Conserv. 31, 149–159. (10.1017/S0376892904001262)

[29] Sweatman H, Cheal A, Emslie M, Johns K, Jonker M, Miller I, Osborne K 2015 *Effects of marine park zoning on coral reefs of the Capricorn-Bunker Group: report on surveys in October 2015*. Cairns, Australia: Reef and Rainforest Research Centre.

[30] Emslie MJ et al 2015 Expectations and outcomes of reserve network performance following re-zoning of the Great Barrier Reef Marine Park. Curr. Biol. 25, 983–992. (10.1016/j.cub.2015.01.073) 25819564

[31] Espinoza M, Cappo M, Heupel MR, Tobin AJ, Simpfendorfer CA 2014 Quantifying shark distribution patterns and species-habitat associations: implications of marine park zoning. PLoS ONE 9, e106885 (10.1371/journal.pone.0106885) 25207545 PMC4160204

[32] Russ GR, Cheal AJ, Dolman AM, Emslie MJ, Evans RD, Miller I, Sweatman H, Williamson DH 2008 Rapid increase in fish numbers follows creation of world's largest marine reserve network. Curr. Biol. 18, R514–R515. (10.1016/j.cub.2008.04.016) 18579091

[33] Catano LB, Rojas MC, Malossi RJ, Peters JR, Heithaus MR, Fourqurean JW, Burkepile DE 2016 Reefscapes of fear: predation risk and reef heterogeneity interact to shape herbivore foraging behaviour. J. Anim. Ecol. 85, 146–156. (10.1111/1365-2656.12440) 26332988

[34] Rizzari JR, Frisch AJ, Hoey AS, McCormick MI 2014 Not worth the risk: apex predators suppress herbivory on coral reefs. Oikos 123, 829–836. (10.1111/oik.01318)

[35] Sweatman H, Robertson DR 1994 Grazing halos and predation on juvenile Caribbean surgeonfishes. Mar. Ecol. Prog. Ser. 111, 1–6. (10.3354/meps111001)

[36] Valentine JF, Heck KL 2005 Perspective review of the impacts of overfishing on coral reef food web linkages. Coral Reefs 24, 209–213. (10.1007/s00338-004-0468-9)

[37] Andréfouët S, Muller-Karger FE, Hochberg EJ, Hu C, Carder KL 2001 Change detection in shallow coral reef environments using Landsat 7 ETM+ data. Remote Sens. Environ. 78, 150–162. (10.1016/S0034-4257(01)00256-5)

[38] Hay ME 1984 Patterns of fish and urchin grazing on Caribbean coral reefs: are previous results typical? Ecology 65, 446–454. (10.2307/1941407)

[39] Madin JS, Madin EMP 2015 The full extent of the global coral reef crisis. Conserv. Biol. 29, 1724–1726. (10.1111/cobi.12564) 26219479

[40] Anonymous. 2014 Nanosats are go! See www.economist.com/technology-quaterly/2014/06/07/nanosats-are-go.

[41] Sweeting M 2018 Modern small satellites—changing the economics of space. Proc. IEEE 106, 343–361. (10.1109/JPROC.2018.2806218)

