# Supplementary material for: Marine reserves shape seascapes on scales visible from space
Source: Proc Biol Sci. 2019 Apr 24;286(1901):20190053. doi: 10.1098/rspb.2019.0053 (PMC6501923; doi:10.1098/rspb.2019.0053)
Supplement: Supplementary information [file rspb20190053supp1.docx]

**Supplementary Information**

**Supplementary tables**

**Table S1**. Generalized linear model (function *glm* from *R* package *stats*) used to quantify the relationship between grazing halo presence and reserve age. Reserve age was the only variable remaining in the model following model selection procedures (Table 1; described in Zuur *et al.* [1]). Nagelkerke's R^2^ = 0.072 (calculated from function *NagelkerkeR2* in *R* package *fmsb*).

|  | Estimate | Std.Error | z.value | p.value |
| --- | --- | --- | --- | --- |
| (Intercept) | -1.253 | 0.472 | -2.655 | 0.008 |
| Reserve age | 0.036 | 0.019 | 1.935 | **0.053** |

**Table S2.** Linear mixed-effects model fit by maximum likelihood (function *lme* from *R* package *nlme*) used to quantify relationships between grazing halo size (i.e., width) and four key predictor variables. Because grazing halo and patch reef size data are at the within-whole reef spatial scale, patch reef area and whole reef ID were included as nested random factors within the model. All other factors, as well as patch reef area, were included as fixed factors. Only those variables remaining in the model following model selection procedures (Table 1; described in Zuur *et al.* [1]) are shown. Marginal R^2^ = 0.482 and conditional R^2^ = 0.617 (calculated from function *r.squaredGLMM* in *R* package *MuMIn*).

(a)

|  | Value | Std.Error | DF | t.value | p.value |
| --- | --- | --- | --- | --- | --- |
| (Intercept) | -42.997 | 18.238 | 185.000 | -2.358 | 0.019 |
| Patch reef area | 0.210 | 0.042 | 185.000 | 5.040 | **0.000** |
| Reserve age | -0.002 | 0.002 | 185.000 | -1.079 | 0.282 |
| Sea surface temperature | 0.145 | 0.061 | 21.000 | 2.384 | **0.027** |
| Chlorophyll *a* concentration | -0.001 | 0.047 | 21.000 | -0.014 | 0.989 |

**Table S3.** Species list of all fishes and macroinvertebrates observed in Heron Island lagoon

video surveys. Columns “Reef” and “Halo” denote presence/absence in these habitats

(1 = present; 0 = absent). Prefix “Un.” refers to unidentified species within a taxon.

| **Species** | **Reef** | **Halo** |
| --- | --- | --- |
| *Abudefduf bengalensis* | 1 | 0 |
| *Abudefduf sexfasciatus* | 1 | 1 |
| *Abudefduf sordidus* | 1 | 1 |
| *Acanthochromis polyacanthus* | 1 | 1 |
| *Acanthurus auranticavus* | 1 | 1 |
| *Acanthurus blochii* | 1 | 0 |
| *Acanthurus grammoptilus* | 1 | 1 |
| *Acanthurus nigricauda* | 1 | 0 |
| *Acanthurus xanthopterus* | 1 | 1 |
| *Achamia leai* | 1 | 0 |
| *Aetobates narinari* | 1 | 1 |
| *Amblyeleotris sp.* | 0 | 1 |
| *Amblyglyphidodon curacao* | 1 | 0 |
| *Amblygobius bynoensis* | 1 | 0 |
| *Amblygobius phalaena* | 1 | 1 |
| *Amblygobius sp.* | 0 | 1 |
| *Amphiprion akindynos* | 1 | 0 |
| *Amphiprion melanopus* | 1 | 0 |
| *Anampses geographicus* | 1 | 0 |
| *Apogon doederleini* | 1 | 0 |
| *Apogon* sp. | 1 | 0 |
| Un. Belonidae | 1 | 1 |
| Un. Blenniidae | 1 | 1 |
| *Caesio teres* | 1 | 1 |
| *Canthigaster valentini* | 1 | 0 |
| *Carangoides* sp. | 1 | 1 |
| *Caranx ignobilis* | 0 | 1 |
| *Caranx* sp*.* | 0 | 1 |
| *Carcharhinus melanopterus* | 1 | 1 |
| *Cephalopholis* sp. | 1 | 0 |
| *Chaetodon auriga* | 1 | 1 |
| *Chaetodon flavirostris* | 1 | 0 |
| *Chaetodon lunula* | 1 | 0 |
| *Chaetodon melannotus* | 1 | 0 |
| *Chaetodon plebeius* | 1 | 0 |
| *Chaetodon rainfordi* | 1 | 1 |
| *Chaetodon vagabundus* | 1 | 0 |
| *Chanos chanos* | 0 | 1 |
| *Cheilinus fasciatus* | 1 | 0 |
| *Cheilinus trilobatus* | 1 | 0 |
| *Chelmon rostratus* | 1 | 0 |
| *Choerodon* sp*.* | 1 | 0 |
| *Chromis nitida* | 1 | 0 |
| *Chromis ternatensis* | 1 | 0 |
| *Chrysiptera cyanea* | 1 | 0 |
| *Chrysiptera rollandi* | 1 | 0 |
| *Coris batuensis* | 1 | 0 |
| *Coris gaimardi* | 1 | 0 |
| *Ctenochaetus striatus* | 1 | 0 |
| *Dascyllus aruanus* | 1 | 0 |
| *Diodon histrix* | 0 | 1 |
| *Dischistodus melanotus* | 1 | 1 |
| *Dischistodus perspicillatus* | 1 | 1 |
| *Echeneis naucrates* | 1 | 1 |
| *Fistularia* sp*.* | 0 | 1 |
| Un. Flatfish | 0 | 1 |
| *Galeocerdo cuvier* | 0 | 1 |
| *Gerres oyena* | 1 | 1 |
| *Gnathodon speciosus* | 0 | 1 |
| *Gymnothorax javanicus* | 1 | 0 |
| *Hemigyminus melapterus* | 1 | 0 |
| *Heniochus acuminatus* | 1 | 0 |
| *Kyphosus* sp. | 1 | 1 |
| *Labroides dimidiatus* | 1 | 1 |
| *Lethrinus* sp*.* | 1 | 1 |
| *Lutjanus carponotatus* | 1 | 1 |
| *Lutjanus fulviflamma* | 1 | 0 |
| *Lutjanus lemniscatus* | 1 | 0 |
| *Monotaxis grandoculis* | 1 | 0 |
| *Naso annulatus* | 1 | 0 |
| *Naso unicornis* | 1 | 1 |
| *Negaprion acutidens* | 1 | 1 |
| *Neoglyphidodon melas* | 1 | 0 |
| *Neoglyphidodon polyacanthus* | 1 | 0 |
| *Ogilbina queenslandie* | 1 | 0 |
| *Ostracion cubicus* | 1 | 0 |
| *Parapercis* sp*.* | 1 | 1 |
| Un. Parrotfish | 1 | 1 |
| Un. Pipefish | 1 | 0 |
| *Platax sp*. | 0 | 1 |
| *Plectorhinchus albovittatus* | 1 | 1 |
| *Plectorhinchus* sp*.* | 1 | 1 |
| *Plectropomus sp*. | 1 | 0 |
| *Pomacanthus sextriatus* | 1 | 0 |
| *Pomacentrus amboinensis* | 1 | 0 |
| *Pomacentrus australis* | 1 | 0 |
| *Pomacentrus molluccensis* | 1 | 1 |
| *Pomacentrus* sp. | 0 | 1 |
| *Ptereleotris* sp. | 1 | 1 |
| *Rachycentrum canadum* | 0 | 1 |
| Un. Sardines | 0 | 1 |
| *Scolopsis bilineatus* | 1 | 1 |
| *Scolopsis margaritifer* | 1 | 0 |
| *Scolopsis monogramma* | 1 | 1 |
| *Scolopsis taeniopterus* | 1 | 0 |
| Un. Sea cucumber | 1 | 1 |
| Un. Sea Turtle | 1 | 1 |
| Un. Ray | 0 | 1 |
| *Siganus canaliculatus* | 1 | 1 |
| *Siganus doliatus* | 1 | 1 |
| *Siganus fuscescens* | 1 | 0 |
| *Siganus lineatus* | 1 | 0 |
| *Siganus puellus* | 1 | 0 |
| *Siganus punctatissimus* | 1 | 1 |
| *Sphyraena* sp. | 1 | 1 |
| Un. Squids | 0 | 1 |
| *Stegastes apicalis* | 1 | 0 |
| *Stegastes* sp. | 1 | 0 |
| Un. Stingray | 1 | 1 |
| *Symphorus nematophorus* | 1 | 1 |
| *Thalassoma lunare* | 1 | 1 |
| *Thalassoma hardwicke* | 1 | 1 |
| *Thalassoma lunare* | 1 | 1 |
| *Thalassoma* sp*.* | 0 | 1 |
| *Trachinocephalus myops* | 0 | 1 |
| *Triaenodon obesus* | 1 | 0 |
| Un. Labrids | 1 | 1 |
| Un. Pomacentrid | 1 | 1 |
| Un. Sand goby | 0 | 1 |
| Un. Shrimp goby | 0 | 1 |
| *Valenciennea longipinnis* | 1 | 1 |
| *Zebrasoma veliferum* | 1 | 1 |

**Supplementary figures**

**Figure S1. Abundance of halo-feeding herbivorous species and their predators in nominally fished (yellow zone) and unfished (green zone) portions of Heron Island lagoon.** Although the yellow zone portion of Heron Island lagoon can legally be fished (it is a “Conservation Park Zone” with limited fishing allowed), the geomorphology of the lagoon means that access to it is difficult to navigate and strongly tidally-dependent. As a result, the level of fishing pressure within the lagoon is minimal (Heron Island Research Station manager, *pers. comm.*). Bars are means (+/- SE) for all patch reefs/halos within Heron Island lagoon. Fifteen of our 22 patch reefs are located in the protected, unfished section (green zone) of Heron Island lagoon, whereas the remaining seven are in the fished section (yellow zone). Higher camera numbers indicate farther distances into the halo from patch reef (camera 1 = 0 m, on reef; camera 2 = 2.5 m from reef; camera 3 = 7.5 m from reef). Herbivores include only those species observed to feed in the halo at any reef (i.e., *Acanthurus* spp., *Signaus* spp., *Zebrasoma veliferum*, and *Naso unicornis*). Predators include only jacks (family Carangidae) and sharks, whose large body size renders them capable of consuming adults of all halo-feeding herbivore species.

**Supplementary experimental procedures**

*In-situ surveys*

Grazing halos were surveyed within the GBR’s Heron Island lagoon (adjacent to Heron Island; 23° 27’ S, 151° 55’ E) at each of 22 replicate patch reefs. Study site characteristics are described in Madin et al. [2]. In-situ grazing assays and algal canopy height measurements were conducted at a subset of three of these patch reefs/halos in July 2010 following the protocol outlined in Madin et al. [2]. For our 2013 camera trap surveys, a marker was positioned at a distance of 3 m in front of each camera to mark the end of the observation area. The camera’s field of view was 5.6m wide. Recording times ranged from 119 to 285 min (mean ± SD: 215.49 ± 48.6 min) per camera. Herbivorous non-cryptic invertebrates (e.g., urchins) were not included in analyses because none were observed in our surveys. Other non-fish herbivores (e.g., turtles) were likewise not included because they appeared in negligible numbers (N = 6 observations) within our surveys and were never observed to feed on the benthic algae that forms the grazing halos in this system, despite it being a potential food source for them.

*Details of Heron lagoon species interactions*

The effect that herbivores will have on the benthos may depend on the herbivore’s diet preferences, the nature of the habitat’s structure, the herbivore’s need for and use of shelter, and the location, type and extent of constraints on the herbivore’s spatial foraging patterns. The algae that constitutes the ‘algal meadows’ atop the sandy matrix within which the halos are found consists largely of a mix of *Enteromorpha spp*. (synonimised now under genus *Ulva*) and *Cladophora spp*. (both green alga), *Hincksia spp*. (a small, fine brown algae), and various other species to lesser degrees (G. Diaz-Pulido, *pers. comm.* based on specimens collected from the study site). This algal matrix provides a palatable and likely nutritious mix of species that both grazers and browsers are likely to consume. The at times lush ‘meadows’ of this algal matrix found at and beyond the edge of the halos should thus provide a strong nutritional incentive for herbivorous fishes to travel off the reef to consume it. This assertion is supported by our grazing assay conducted using this algal matrix (Fig. 1B, light green bars) which shows that algae placed within the halo is rapidly consumed, particularly in areas closest to the patch reefs.

In terms of foragers, the herbivore guild is dominated by the functional groups of grazers, scrapers, and detritivores (e.g., including some Acanthurids, Pomacanthids, Labrids/Scarids, and some Siganids), or those species that feed largely on epilithic algal turfs growing on hard substratum [3]. Browsers, by contrast, consistently feed on fleshy macroalgae [3] or seagrass (e.g., including some Acathurids [primarily genus Naso], Ephippids, Kyphosids, some Labrids/Scarids, and some Siganids) and make up the remaining component of the herbivore guild. We observed many individuals (often in groups of 5 or more) of both the former and the latter groups on our study reefs and/or within the bare, halo areas themselves (i.e., adjacent to or away from patch reefs). We observed a subset of these groups actively feeding on benthic algae in the halo (*Acanthurus* spp., *Signaus* spp., *Zebrasoma veliferum*, and *Naso unicornis*). While larger-bodied adults of these functional groups will not generally remain within the shelter of the reef matrix during the day, they will quickly and predictably retreat to these shelters when confronted with real or perceived risk (E. Madin, *pers. obs.*). This suggests that regardless of where their primary food source is located (i.e., on or adjacent to the reef), all individuals of these groups likely benefit to some extent from remaining in close proximity to the reef’s shelter.

*Satellite imagery surveys*

The entire ~2300 km length of the Great Barrier Reef (GBR) was surveyed for grazing halos using high spatial resolution satellite imagery accessed through Google Earth Pro (GEP). The GEP platform includes a mix of medium (i.e., ~30 m) to very high (i.e., <3 m) spatial resolution imagery from a variety of imagery providers and satellites, both non-commercial (i.e., the US Geological Survey’s *Landsat*) and commercial (e.g., DigitalGlobe’s *IKONOS*, *Quickbird*, *Worldview-1* and *Worldview-2*; SpotImage’s *SPOT* satellites; former GeoEye’s *GeoEye-1*). The resolution of imagery in GEP varies depending on the source of the data, and it is not possible from within this platform to determine the specific satellite from which a given scene (i.e., panel) of imagery was acquired. The imagery used in the study was collected between 2000 and 2012, with the frequency distribution of the imagery following no clear pattern over time.

Halo visibility was variable depending on image quality (e.g., image spatial resolution, cloud cover, sea surface reflection/sun glint) and background benthic algal density. For whole reefs where image quality was sufficient to allow identification of individual grazing halos where they existed (i.e., imagery was taken at < 3m spatial resolution, it provided a clear view of the reef’s benthic features, and there was sufficient benthic algal coverage to permit the formation of halos), the entire reef was visually scanned by a single human annotator from north to south for the presence of grazing halos surrounding patch reefs. For whole reefs containing halos, the width of ten of the most visible halos and the areas of their respective interior patch reefs were measured manually by the same annotator. On the small subset of whole reefs where halos were visible but fewer than ten could be measured, all halos were measured. The area of halos and the patch reefs they encircled were measured by manually tracing the outline of the halo and the patch reef using Google Earth Pro’s polygon measurement tool. When a halo being outlined included part of a neighboring patch reef or was partially merged with a neighboring halo, the outline was drawn around a visual interpolation of what the halo might look like in isolation (i.e., generally following the reef’s contour and at a width similar to the rest of the halo). If the reef or halo was not clearly visible because of image quality, or if the reef was surrounded by sand, by sand on one side and algae on the other, or by a halo that extended less than halfway around the reef, no halo was outlined for that reef. Only halos which were clearly visible and clearly defined, within the limits of the image resolution, were outlined. For each whole reef, we also searched GEP’s historical imagery for the presence of halos in previous years and measured up to ten halos for each image date. Once reefs with either a) poor satellite image quality that did allow us to visually discern whether or not halos were present or b) lack of suitable patch reef habitat around which halos can potentially form were removed from the dataset, 214 whole reefs (each containing hundreds to thousands of patch reefs) were used in analyses.

Sea surface temperature (SST) data are derived from the UK Met Office’s Operational SST and Sea Ice Analysis (OSTIA) system dataset. These data are long-term averaged SST data for the GBR in Kelvin units. The OSTIA system produces a foundation SST estimate at an output grid resolution of 1/20° (~6km), although the smallest analysis feature resolution is based on the correlation length scale of 10 km. Details of this dataset can be found in Donlon *et al.* [4].

Chlorophyll a concentration (Chl a) data are derived from MERIS (the MEdium Resolution Imaging Spectrometer; <https://earth.esa.int/web/guest/missions/esa-operational-eo-missions/envisat/instruments/meris>). These data are monthly averages from 2003-2011, based on daily chlorophyll concentrations, and are spatially interpolated to ~9 km. The algorithm used to extract these chlorophyll data is optimized for coastal areas and takes into account high turbidity and bottom reflectance, and is thus presumed to be reflective of phytoplankton chlorophyll values. Despite the fact that algae and seagrass are best discerned by shorter wavelengths than the shortest (390nm) MERIS band, and thus the assumption that these data reflect phytoplankton chlorophyll values is likely to be correct, chlorophyll values for shallow areas such as ours should always be interpreted with some caution.

*Variable selection*

A subset of reefs within the Great Barrier Reef (GBR) are regularly surveyed to determine population sizes of a subset of herbivorous and predatory fishes [5], however there is little overlap (N locations = 6) between the sites that dataset covers and those for which high-resolution satellite data exists and grazing halos were observed. This lack of spatial overlap precludes reliable analyses of grazing halo presence or size based on in-situ herbivore or predator population data. Studies from around the world [6,7] indicate that fisheries-targeted species, including herbivores and predators, tend to increase in number as reserves age (predators consistently so) in systems where they are targeted by fisheries. We therefore used marine reserve age as a proxy variable likely to indicate relative levels of herbivore and/or predator density over the large spatial scale of our study system. Distance from mainland was initially included, but subsequently excluded, because it showed a strong correlation with SST (discussed below).

Other variables included in the initial model were those hypothesized to affect grazing halo presence and/or width in another way. SST was included as a proxy for metabolic rates of herbivores, which we hypothesized could influence grazing rates [8]. Latitude, both a presumed proxy for metabolic rates of herbivores (via temperature) and a proxy for the biogeographic gradient over which the GBR occurs, was initially included but subsequently excluded due to its strong correlation with SST, which we deemed more relevant to our predictions. Chl a was included as a proxy for primary productivity, which we hypothesized could influence halo occurrence/width via primary producer growth rates. We chose to use Chl *a* rather than a metric of net primary productivity (NPP) that integrates Chl *a* and temperature, such as the standard Vertically Generalized Production Model (VGPM), in order to allow us to explicitly include temperature in our model as a stand-alone predictor variable given its potential effect on herbivore physiology. Image season (i.e., the time of year in which the satellite image was captured) was initially included to account for seasonal differences in algal standing stock and thus halo visibility, but it was subsequently dropped due to data loss because a large portion of the dataset’s images’ meta-data contained only the year, not the month or season, of capture. Lastly, in the analysis of halo width, the additional variable of patch reef area was included because it was hypothesized that for either geometric and/or biological reasons, this factor could influence grazing halo size.

**Supplemental references**

1. Zuur AF, Ieno EN, Walker NJ, Saveliev AA, Smith GM. 2009 *Mixed effects models and extensions in ecology with R*. New York, NY: Springer.

2. Madin EMP, Madin JS, Booth DJ. 2011 Landscape of fear visible from space. *Sci. Rep.* **1**, 14. (doi:10.1038/srep00014)

3. Green AL, Bellwood DR. 2009 Monitoring functional groups of herbivorous reef fishes as indicators of coral reef resilience – A practical guide for coral reef managers in the Asia Pacific region. *IUCN Work. Gr. Clim. Chang. Coral Reefs*.

4. Donlon CJ, Martin M, Stark J, Roberts-Jones J, Fiedler E, Wimmer W. 2012 The Operational Sea Surface Temperature and Sea Ice Analysis (OSTIA) system. *Remote Sens. Environ.* **116**, 140–158. (doi:10.1016/j.rse.2010.10.017)

5. Emslie MJ *et al.* 2015 Expectations and outcomes of reserve network performance following re-zoning of the Great Barrier Reef Marine Park. *Curr. Biol.* **25**, 1–10. (doi:10.1016/j.cub.2015.01.073)

6. Friedlander AM, Golbuu Y, Ballesteros E, Caselle JE, Gouezo M, Olsudong D, Sala E. 2017 Size, age, and habitat determine effectiveness of Palau’s Marine Protected Areas. *PLoS One* **12**, 1–18. (doi:10.1371/journal.pone.0174787)

7. Edgar GJ *et al.* 2014 Global conservation outcomes depend on marine protected areas with five key features. *Nature* **506**, 216–20. (doi:10.1038/nature13022)

8. Floeter SR, Behrens MD, Ferreira CEL, Paddack MJ, Horn MH. 2005 Geographical gradients of marine herbivorous fishes: Patterns and processes. *Mar. Biol.* **147**, 1435–1447. (doi:10.1007/s00227-005-0027-0)
